# Thrombotic thrombocytopenia After Sinopharm BBIBP‐CorV COVID‐19 vaccination

**DOI:** 10.1002/rth2.12750

**Published:** 2022-06-21

**Authors:** Rezvan Hosseinzadeh, Mohammad Barary, Hamed Mehdinezhad, Terence T. Sio, Florian Langer, Sahar Khosravi

**Affiliations:** ^1^ Student Research Committee Babol University of Medical Sciences Babol Iran; ^2^ Student Research Committee, Virtual School of Medical Education and Management Shahid Beheshti University of Medical Sciences Tehran Iran; ^3^ Students’ Scientific Research Center (SSRC) Tehran University of Medical Sciences Tehran Iran; ^4^ Department of Internal Medicine, Rouhani Hospital Babol University of Medical Sciences Babol Iran; ^5^ Department of Radiation Oncology Mayo Clinic Phoenix Arizona USA; ^6^ Zentrum für Onkologie, II. Medizinische Klinik und Poliklinik, Universitätsklinikum Eppendorf Hamburg Germany; ^7^ HSCT and Cancer Research Center of Shariati Hospital Tehran University of Medical Sciences Tehran Iran

**Keywords:** COVID‐19, SARS‐CoV‐2, Sinopharm, thrombosis, vaccine, VITT

## Abstract

**Background:**

Severe side effects after vaccination with coronavirus disease 2019 (COVID‐19) vaccines are rare but can be fatal. To date, vaccine‐induced immune thrombotic thrombocytopenia (VITT) cases have been reported after injection of mRNA and adenoviral vectors COVID‐19 vaccines. Here, we report the second suspected case of VITT after vaccination with the Sinopharm vaccine, an inactive vaccine.

**Key Clinical Question:**

The Key Clinical Question was to determine whether inactivated COVID‐19 vaccines could induce VITT and how to diagnose and treat such cases.

**Clinical Approach and Conclusions:**

Our patient developed deteriorating symptoms the day after vaccination and was admitted to the emergency department on day 5 after vaccination. After performing laboratory analysis, thrombosis with thrombocytopenia was suggested, further confirmed by highly positive anti‐heparin–platelet factor 4 antibodies assay and color Doppler ultrasonography. He was then treated with high‐dose intravenous immunoglobulin, corticosteroid, and nonheparin anticoagulant.

**Essentials**

- Vaccine‐induced immune thrombotic thrombocytopenia (VITT) has been reported after the coronavirus disease 2019 (COVID‐19) vaccines.
- This is the second report of VITT after vaccination with the BBIBP‐CorV COVID‐19 vaccine.
- VITT is diagnosed by low platelets and fibrinogen, and high D‐dimer and anti– platelet factor 4 antibodies.
- VITT can be treated with intravenous immunoglobulin, nonheparin anticoagulants, and high‐dose corticosteroids

## 1 BACKGROUND

Coronavirus disease 2019 (COVID‐19), caused by severe acute respiratory syndrome coronavirus 2 (SARS‐CoV‐2),^1^ has had a significant impact on public health, with more than 172 million confirmed cases and more than 3 million deaths worldwide so far (as of June 6, 2021).^2^ Because of the global COVID‐19 pandemic, several vaccines against the SARS‐CoV‐2 infection were expediently developed, and seven were approved by the World Health Organization,^1^ one of which is the Sinopharm COVID‐19 (BBIBP‐CorV) vaccine.^3^ This inactivated SARS‐CoV‐2 vaccine was made from the HB02 strain.^4^ The common side effects reported following COVID‐19 vaccination were mostly self‐limiting, quickly resolving local reactions. Nevertheless, recently, some reports of thrombosis and thrombocytopenia after the administration of certain COVID‐19 vaccines were reported, especially the adenoviral vector types, that is, Oxford/AstraZeneca (ChAdOx1)^5^ and Johnson & Johnson (Janssen).^6^

Venous thrombosis and thrombocytopenia were initially observed after the first dose of the ChAdOx1 nCoV‐19 (Oxford‐AstraZeneca) vaccine.^5^, ^7^ Immunogenic thrombosis caused by ChAdOx1 nCoV‐19 primarily affects cerebral veins and sinuses for unknown reasons.^8^ Studies on the side effects of the ChAdOx1 nCOV‐19 vaccine suggested that it can induce an immune reaction similar to that of heparin‐induced thrombocytopenia (HIT) by producing autoantibodies to PF4–polyanion complex, which activates the platelets, thus, causing thrombosis and thrombocytopenia.^5^ Moreover, cases of disseminated intravascular coagulation and extensive thrombosis were observed following injection of the first dose of Ad26.COV2.S (Johnson & Johnson) vaccine.^6^, ^7^

HIT, a prothrombotic disorder, typically occurs 4 to 14 days after initiation of heparin anticoagulation. In contrast, another type of prothrombotic disease caused by platelet activation in the absence of previous heparin exposure is spontaneous HIT, a type of autoimmune HIT (aHIT).^9^ After injecting some COVID‐19 vaccines, a rare complication similar to aHIT occurred, which was subsequently named vaccine‐induced immune thrombotic thrombocytopenia (VITT).^10^, ^11^ Devi et al^12^ recently described the first case of VITT following an inactivated (Sinopharm) COVID‐19 vaccine. This report presents the second suspected case of VITT associated with the Sinopharm BBIBP‐CorV COVID‐19 vaccine.

## 2 CASE REPORT

An 85‐year‐old Persian man with a 5‐day history of fever, chills, abdominal pain, nausea, and peripheral edema was admitted to the emergency department. The patient had received the first dose of the Sinopharm BBIBP‐CoV vaccine, and 1‐day after vaccination, he was referred to an outpatient clinic due to fever and chills, for which his symptoms were treated conservatively. He continued to have fever and chills, which persisted for 3 days, and he became more stuporous and difficult to arouse. On day 5 after vaccination, the patient presented to the emergency department after a syncopal episode. His past medical history was uneventful except for ischemic heart disease, for which he took 80 mg of aspirin daily. The patient and his companions did not mention the history of hospitalization during the past year, but the patient underwent angiography 3 years ago due to ischemic heart disease, and his platelet counts in the test he had 2 months ago were 337 000/μL. He also did not mention any allergies to medications. On physical examination, he presented 3+ pitting peripheral edema and left upper quadrant (LUQ) tenderness.

His laboratory results on admission are summarized in Table 1. The patient’s albumin level was 3.9 g/dL on the 10th day after vaccination. The patient received a SARS‐CoV‐2 polymerase chain reaction assay of the nasopharyngeal swab and high‐resolution computed tomography(CT) scan, both negative for having an active COVID‐19 infection. Anti–spike protein and anti‐nucleocapsid protein antibodies of SARS‐CoV‐2 were positive, indicating the previous infection. CT venography was performed for the patient, which was normal. The cerebral venous sinus thrombosis (CVST) diagnosis was ruled out as the patient did not manifest symptoms, such as severe headache or loss of consciousness.

**TABLE 1:** Clinical and laboratory characteristics of the patient on admission, within the hospitalization, and after the 1‐week follow‐up periods

Characteristic	Reference value	Findings		
		On admission (5th day after vaccination)	Hospitalization period (5th to 15th day after vaccination)	Follow‐up period
Time from vaccination to admission (day)	5			–
WBC count (per μL)	4000‐10 500	2800	2800^1^	10 300
Platelet count (per μL)	150 000‐400 000	51 000	15 000^1^	396 000
Hemoglobin (g/dL)	13‐17	12	9.4^1^	12
D‐dimer (ng/mL)	<200	>3200	>3200^2^	495
Fibrinogen (mg/dL)	200‐400	172	172^1^	260
INR		1	1^2^	1
PTT (s)	25‐45	28	28^2^	30
LDH (U/L)	<480	816	816^2^	275
CRP (mg/L)	<10	65	89^2^	14
ESR (mm/h)	<20	50	60^2^	31
SARS‐CoV‐2 antibody test results				
Spike protein		Positive		Positive
Nucleocapsid protein		Positive		Positive
SARS‐CoV‐2 RT‐PCR test		Positive		Negative

*Note*: Laboratory findings under the hospitalization period column represent ^1^nadir or ^2^peak values of that specific paraclinical test.

Abbreviations: CRP, C‐reactive protein; ESR, erythrocyte sedimentation rate; IHD, ischemic heart disease; INR, international normalized ratio; LDH, lactate dehydrogenase; PTT, partial thromboplastin time; RT‐PCR, reverse transcriptase polymerase chain reaction; SARS‐CoV‐2, severe acute respiratory distress syndrome coronavirus 2; WBC, white blood cell.

**FIGURE 1:**
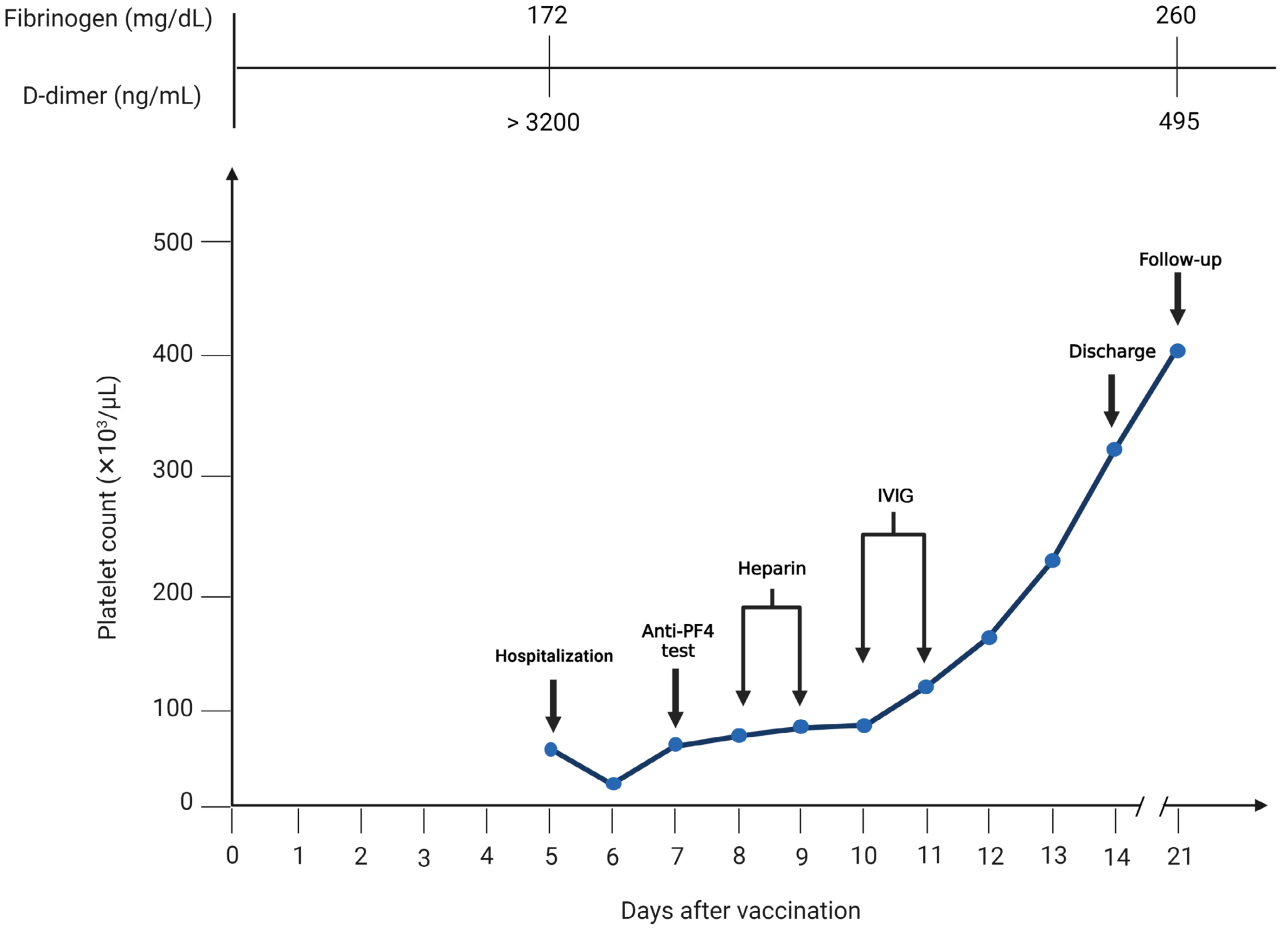
Platelet count in the days after vaccination. The patient was hospitalized on the 5th day after vaccination. An anti‐PF4 test was performed on the 7th day after vaccination. Due to the clinical suspicion of thrombosis, heparin was prescribed to the patient until the test result was prepared. The patient’s heparin was discontinued on the 10th day after vaccination due to a positive anti‐PF4 test result, and IVIG was administered instead. The patient was discharged from the hospital on the 14th day after vaccination, after 10 days of hospitalization. The patient went to the clinic for follow‐up 7 days after discharge. anti‐PF4, anti–platelet factor 4; IVIG, intravenous immunoglobulin

The patient was treated with intravenous analgesics, intravenous antibiotics, and 5000 units of unfractionated heparin subcutaneously twice daily for prophylaxis of venous thromboembolism. Nevertheless, 8 days after vaccination, no changes in his clinical features were observed, and the platelet level remained low (Table 1; Figure 1). Also, because of his LUQ tenderness, abdominopelvic ultrasonography was performed without pathologic findings. Nonetheless, because of his persistent abdominal pain, low platelet counts (51 000/μL), and high D‐dimer levels (>3200), a suspicion of splanchnic vein thrombosis (SVT) was considered, and abdominal color Doppler ultrasonography was performed. The inferior vena cava and portal and hepatic veins were normal, but there was evidence of thrombosis in the splenic vein with mild splenomegaly. Based on the VITT‐adapted 4Ts scoring system, the incidence of VITT in this patient was highly probable.^13^ Thus, platelet factor 4 (PF4)‐heparin (MyBiosource, Inc., San Diego, CA, USA) and PF4 (Thermo Fisher Scientific, Waltham, MA, USA) ELISA kits were used to measure direct antibody binding, with antibody binding measured by a secondary antihuman IgG,^5^ resulting in a highly positive (optical density [OD] = 2.178; the reference values for strong, intermediate, or weak positive are OD ≥2.00, 1.00‐1.99, or 0.50‐0.99, respectively). Nine days after vaccination, treatment, as recommended for VITT (9), was started, and the patient received 40 mg of dexamethasone IV daily for 4 days, intravenous immunoglobulin (IVIG) (1 g/kg for 2 days), and rivaroxaban (15 mg twice a day). After that, the platelet counts steadily recovered (Figure 1), and he was discharged with rivaroxaban 15 mg daily 10 days later. The patient was seen in the follow‐up clinic 1 week later and was noted to have complete symptom relief, except for 1+ peripheral edema and normalization of his laboratory values (Table 1).

## 3 DISCUSSION

Due to the COVID‐19 pandemic and its rapid spread, several vaccines have been developed. As a rapid adaptation of these vaccines is necessary, phase 4 clinical trials with long‐term follow‐up and more in‐depth data on their adverse events are unavailable. National and global regulatory agencies issued emergency use authorization for these vaccines based on analyzing the data of phase III trials. Most of the vaccines’ adverse effects are nonsevere, localized, and self‐limiting, improving spontaneously or within a short time. On the other hand, some reports of a rare, severe, life‐threatening side effect of some COVID‐19 vaccines, called VITT, triggered by the vector‐based vaccines were already reported. In the European Union, as of April 4, 2021, about 222 cases of VITT were reported, of which 169 cases had CVST, and 52 were presented with SVT.^14^ The underlying mechanism is still not well characterized, but certain constituents of the vaccine preparations are thought to serve as polyanions, binding to PF4 and triggering an immune response with the formation of platelet‐activating antibodies (16, 17).

Following the report of Devi et al,^12^ this report presents the second case of VITT following vaccination with the first dose of a different type of vaccine, the inactivated virus Sinopharm COVID‐19 (BBIBP‐CorV) vaccine, in an 85‐year‐old with a 5‐day history of fever, chills, abdominal pain, nausea, peripheral edema, and decreased levels of consciousness. Previous studies demonstrated that VITT could manifest with fever, myalgia, headache, fatigue, chills, nausea, epigastric discomfort, headache, reduced consciousness, back pain, stroke symptoms, hemiparesis, dizziness, noted gum bleeding, decreased oral intake, and anorexia.^5^, ^7^, ^11^, ^15^, ^16^ The typical pattern of VITT, thrombocytopenia, low fibrinogen levels, very high D‐dimer, and high titers of anti‐heparin–PF4 antibodies were present in our patient, together with the sonographically confirmed thrombosis in the splenic vein. According to a study by Pavord et al, our case was a probable VITT case because the D‐dimer was >4000 FEU. The patient had thrombosis and thrombocytopenia. The anti‐PF4 antibodies on ELISA were also positive for our patient.^17^ Thus, although the platelet‐activating assay could not be performed in this patient, VITT could not be excluded. Therefore, consistent with existing expert recommendations^8^ and previous studies,^1^, ^5^, ^7^, ^11^, ^15^, ^16^ this patient was treated with nonheparin anticoagulants, IVIG, and steroids. The patient recovered completely after the treatment, and his laboratory results were normalized during the 1‐week follow‐up.

## 4 CONCLUSION

In conclusion, this case report provides evidence of the second suspected case of VITT triggered after the vaccination with the Sinopharm COVID‐19 vaccine. Although this vaccine has a different composition than the vector‐based vaccines, VITT was initially described, and symptoms started after vaccination. Hence, VITT should be considered after other types of COVID‐19 vaccine, and appropriate diagnostic and therapeutic procedures should be performed as recommended for VITT.

## AUTHOR CONTRIBUTIONS

RH and MB: data collection and writing the original draft. HM, TTS, and FL: data collection, helped writing the original draft, and provided substantial revisions to the manuscript’s content. SK: patient care, study supervision, and helped writing the original draft.

## RELATIONSHIP DISCLOSURE

TTS reports that he provides strategic and scientific recommendations as a member of the advisory board and speaker for Novocure, Inc. and also as a member of the advisory board to Galera Therapeutics, which are not in any way associated with the content or disease site as presented in this article. All other authors have no relevant financial interests to be declared.

## INFORMED CONSENT

As required by the Babol University of Medical Sciences ethics committee, an informed consent form was taken from the patient to report the case.

## References

[1] Scully M , Singh D , Lown R , et al. Pathologic antibodies to platelet factor 4 after ChAdOx1 nCoV‐19 vaccination. N Engl J Med. 2021;384:2202‐2211. 33861525 10.1056/NEJMoa2105385 PMC8112532

[2] World Health Organization . Bangladesh: WHO coronavirus disease (COVID‐19) dashboard with vaccination data | WHO coronavirus (COVID‐19) dashboard with vaccination data. World health Organization 2021: 1–5.

[3] World Health Organization (WHO) . World Health Organisation Extraordinary meeting of the Strategic Advisory Group of Experts on Immunization (SAGE) – 29 April 2021. Available at: Who.2021.

[4] Wang H , Zhang Y , Huang B , et al. Development of an inactivated vaccine candidate, BBIBP‐CorV, with potent protection against SARS‐CoV‐2. Cell. 2020;182:713‐721.e9. 32778225 10.1016/j.cell.2020.06.008 PMC7275151

[5] Greinacher A , Thiele T , Warkentin TE , Weisser K , Kyrle PA , Eichinger S . Thrombotic thrombocytopenia after ChAdOx1 nCov‐19 vaccination. N Engl J Med. 2021;384:2092‐2101. 33835769 10.1056/NEJMoa2104840 PMC8095372

[6] Schuchat A , Marks P . Joint CDC and FDA statement on Johnson & Johnson COVID‐19 vaccine: the following statement is attributed to Dr. Anne Schuchat, Principal Deputy Director of the CDC and Dr. Peter Marks, director of the FDA's Center for Biologics Evaluation and Research: (U.S.) C for DC and P, (U.S.) C for BE and R, editors. Atlanta, GA; 2021: 1–2.

[7] Muir K‐L , Kallam A , Koepsell SA , Gundabolu K . Thrombotic thrombocytopenia after Ad26.COV2.S vaccination. N Engl J Med. 2021;384:1964‐1965. 33852795 10.1056/NEJMc2105869 PMC8063883

[8] Oldenburg J , Klamroth R , Langer F , et al. Diagnosis and management of vaccine‐related thrombosis following AstraZeneca COVID‐19 vaccination: guidance statement from the GTH. Hamostaseologie. 2021;41(3):184‐189. Erratum in: Hamostaseologie. 2021 May 12. 33822348 10.1055/a-1469-7481

[9] Greinacher A , Selleng K , Warkentin TE . Autoimmune heparin‐induced thrombocytopenia. J Thromb Haemost. 2017;15:2099‐2114. 28846826 10.1111/jth.13813

[10] McGonagle D , De Marco G , Bridgewood C . Differences in venous immunothrombosis in severe COVID‐19 pneumonia and vaccine‐induced thrombotic thrombocytopenia (VITT) – It's in the viral DNA. J Autoimmun. 2021;121:102662. 34051613 10.1016/j.jaut.2021.102662 PMC8133385

[11] Thaler J , Ay C , Gleixner KV , et al. Successful treatment of vaccine‐induced prothrombotic immune thrombocytopenia (VIPIT). J Thromb Haemost. 2021;19:1819‐1822. 33877735 10.1111/jth.15346 PMC8362082

[12] Devi K , Ali N , Nasir N , Mahmood SF . VITT with inactivated SARS‐CoV‐2 vaccine ‐ index case. Hum Vaccin Immunother. 2022;18:2036556. 35254213 10.1080/21645515.2022.2036556 PMC9009890

[13] Warkentin TE , Cuker A . VITT‐adapted 4Ts scoring system. In: Post TedW , ed. UpToDate. UpToDate; 2021.

[14] von Hundelshausen P , Lorenz R , Siess W , Weber C . Vaccine‐induced immune thrombotic thrombocytopenia (VITT): targeting Pathomechanisms with Bruton tyrosine kinase inhibitors. Thromb Haemost. 2021;121:1395‐1399. 33851389 10.1055/a-1481-3039

[15] Dhoot R , Kansal A , Handran C , et al. Thrombocytopenia and splanchnic thrombosis after Ad26.COV2.S vaccination successfully treated with transjugular intrahepatic portosystemic shunting and thrombectomy. Am J Hematol. 2021;96:1180‐1182. 34057234 10.1002/ajh.26258 PMC8212098

[16] Schultz NH , Sørvoll IH , Michelsen AE , et al. Thrombosis and thrombocytopenia after ChAdOx1 nCoV‐19 vaccination. N Engl J Med. 2021;384:2124‐2130. 33835768 10.1056/NEJMoa2104882 PMC8112568

[17] Pavord S , Scully M , Hunt BJ , et al. Clinical features of vaccine‐induced immune thrombocytopenia and thrombosis. N Engl J Med. 2021;385:1680‐1689. 34379914 10.1056/NEJMoa2109908 PMC10662971

